# 3-(1,2-Diphenyl­ethen­yl)-2-phenyl-1*H*-indole

**DOI:** 10.1107/S1600536810039309

**Published:** 2010-10-09

**Authors:** P. A. Abdullah Mahaboob, M. NizamMohideen, G. Bhaskar, P. T. Perumal

**Affiliations:** a Department of Physics, The New College (Autonomous), Chennai 600 014, India; b Organic Chemistry Division, Central Leather Research Institute, Chennai 600 020, India

## Abstract

In the title compound, C_28_H_21_N, the planar pyrrole ring makes dihedral angles of 1.5 (2), 42.4 (2), 65.4 (2) and 79.7 (1)°, with the least squares planes of the four phenyl rings. The mol­ecular structure and crystal packing are stabilized by weak inter- and intra­molecular C—H⋯π inter­actions.

## Related literature

For applications of heteroarenes, see: Ritleng *et al.* (2002 ▶). For their pharmaceutical properties and related reactions, see: Sundberg (1996 ▶); Ferrer *et al.* (2007 ▶); Nair *et al.* (2004 ▶; Sakai *et al.* (2006 ▶, 2008 ▶); Cheng *et al.* (2007 ▶); For standard bond lengths, see: Allen *et al.* (1987 ▶). For bond distances and angles in related structures, see: NizamMohideen *et al.* (2010*a* ▶,*b* ▶).
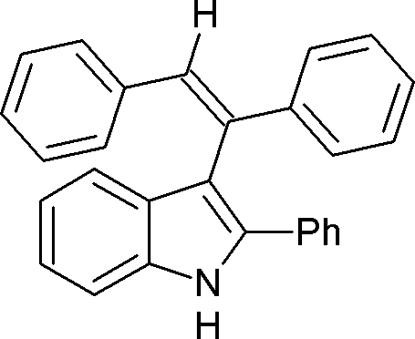


## Experimental

### 

#### Crystal data

C_28_H_21_N
*M* *_r_* = 371.46
Monoclinic, 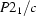

*a* = 11.4227 (6) Å
*b* = 8.6998 (5) Å
*c* = 20.6203 (13) Å
β = 94.413 (4)°
*V* = 2043.1 (2) Å^3^
*Z* = 4
Mo *K*α radiation
μ = 0.07 mm^−1^
*T* = 298 K
0.32 × 0.28 × 0.22 mm

#### Data collection

Bruker Kappa APEXII CCD diffractometer’
Absorption correction: multi-scan (*SADABS*; Bruker, 2004 ▶) *T*_min_ = 0.978, *T*_max_ = 0.985
14625 measured reflections
4674 independent reflections
1701 reflections with *I* > 2σ(*I*)
*R*_int_ = 0.057

#### Refinement

*R*[*F* ^2^ > 2σ(*F* ^2^)] = 0.070
*wR*(*F* ^2^) = 0.221
*S* = 1.01
4674 reflections
266 parameters
H atoms treated by a mixture of independent and constrained refinement
Δρ_max_ = 0.30 e Å^−3^
Δρ_min_ = −0.14 e Å^−3^

### 

Data collection: *APEX2* (Bruker, 2004 ▶); cell refinement: *APEX2* and *SAINT* (Bruker, 2004 ▶); data reduction: *SAINT* and *XPREP* (Bruker, 2004 ▶); program(s) used to solve structure: *SHELXS97* (Sheldrick, 2008 ▶); program(s) used to refine structure: *SHELXL97* (Sheldrick); molecular graphics: *ORTEP-3* (Farrugia, 1997 ▶) and *PLATON* (Spek, 2009 ▶); software used to prepare material for publication: *SHELXL97* and *PLATON*.

## Supplementary Material

Crystal structure: contains datablocks global, I. DOI: 10.1107/S1600536810039309/jj2059sup1.cif

Structure factors: contains datablocks I. DOI: 10.1107/S1600536810039309/jj2059Isup2.hkl

Additional supplementary materials:  crystallographic information; 3D view; checkCIF report

## Figures and Tables

**Table 1:** Hydrogen-bond geometry (Å, °) *Cg*1 and *Cg*2 are the centroids of the N1/C1/C2/C3/C8 and C3–C8 rings, respectively.

*D*—H⋯*A*	*D*—H	H⋯*A*	*D*⋯*A*	*D*—H⋯*A*
C18—H18⋯*Cg*1	0.93	2.92	3.562 (2)	127
C20—H20⋯*Cg*2^i^	0.93	2.92	3.825 (2)	164

Symmetry code: (i) 
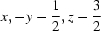
.

**Fig. 1.**
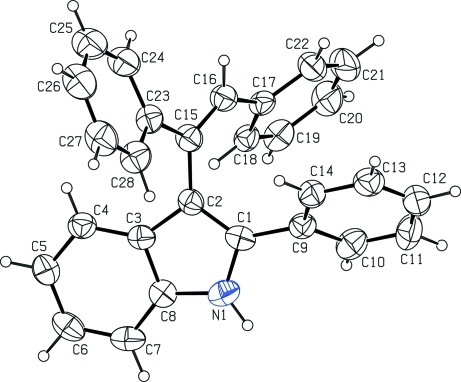
The molecular structure of the title compound with the atom numbering scheme and 50% probability displacement ellipsoids. H atoms are presented as a small spheres of arbitrary radius.

**Fig. 2.**
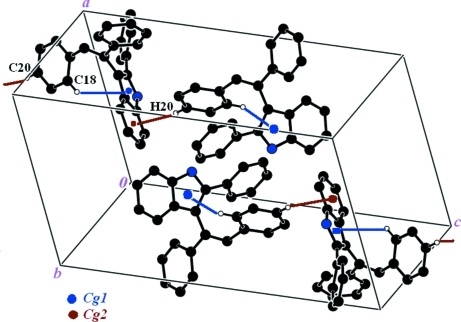
C—H···π interactions (dashed lines) in the title compound. Cg denotes the ring centroid. [Symmetry codes: (i) x,y,z; (ii) x,1/2-y,-1/2+z]

## supplementary crystallographic information

### Comment

The indole ring system exists ubiquitously in natural products, and exhibits biological and pharmaceutical properties (Sundberg, 1996). Ferrer and co-workers reported a systematic investigation on the gold-catalyzed intra- and intermolecular addition of indoles to alkynes (Ferrer *et al.*, 2007). Cheng and co-workers investigated the reaction of indoles with alkynyl alcohols employing platinum as a catalyst (Cheng *et al.*, 2007). Development of heteroarene functionalization has attracted much attention of their wide range of applications such as fluorescent dyes, synthetic analogues of natural products, and pharmaceuticals (Ritleng *et al.*, 2002). There has been considerable interest in the catalytic use of indium(III) halides in organic synthesis (Nair *et al.*, 2004), due to their unique properties such as non-toxicity, stability in air, and water tolerance (Sakai *et al.*, 2006). Indium(III) bromide is known to catalyze intramolecular cyclization of 2-alkynylanilines (Sakai *et al.*, 2008). In continuation of our work in this area, the title compound, C_28_H_21_N, (I) has been prepared and its crystal structure is reported.

In the title compound the pyrrole ring is planar, the maximum deviation from the least squares plane being -0.009 (1)Å for atom N1. The dihedral angle formed by the least squares planes of the pyrrole ring and the four benzene rings is 1.5 (2)° (C3—C8), 42.4 (2)° (C9—C14), 65.4 (2)° (C17—C22) and 79.7 (1)° (C23—C28), respectively. The dihedral angle between the phenyl rings C9—C14 and C23—C28 is 88.5 (2)°.  The dihedral angle between benzene rings C3—C8 and C17—C22 is 66.7 (7)° and between rings C17—C22 and C23—C28 is 87.0 (2)°.   All bond lengths and angles are within normal ranges (Allen *et al.*, 1987) and comparable with those in a previously reported structure (NizamMohideen *et al.*, 2010a,b).  The molecular packing is stabilized by an intra and intermolecular C—H···π interactions (Table 1).

### Experimental

A mixture of diphenylacetylene (2.4 mmol), 2-Phenyl indole (2 mmol), indium tribromide (0.2 mmol) in toluene (4 ml) was stirred at 383° K temperature for 2.5 h. After completion of the reaction as indicated by TLC, the reaction mixture was diluted with water and extracted with ethyl acetate. The combined organic layers were dried over anhydrous Na_2_SO_4_, concentrated *in vacuo* and purified by column chromatography on silica gel (Merck, 100 - 200 mesh) to afford the desired product after crystallization.

### Refinement

H1N was located by a difference fourier map and refined isotropically.  All other H atoms were positioned geometrically, with C—H = 0.93 and N—H = 0.89Å constrained to ride on their parent atoms, with *U*_iso_(H) = *xU*_eq_(C, N), where *x* = 1.5 for methyl H and *x* = 1.2 for all H atoms.

### Crystal data

**Table:**

C_28_H_21_N	*F*(000) = 784
*M**_r_* = 371.46	*D*_x_ = 1.208 Mg m^−^^3^
Monoclinic, *P*2_1_/*c*	Mo *K*α radiation, λ = 0.71073 Å
Hall symbol: -P 2ybc	Cell parameters from 1461 reflections
*a* = 11.4227 (6) Å	θ = 2.5–18.8°
*b* = 8.6998 (5) Å	µ = 0.07 mm^−^^1^
*c* = 20.6203 (13) Å	*T* = 298 K
β = 94.413 (4)°	Block, colourless
*V* = 2043.1 (2) Å^3^	0.32 × 0.28 × 0.22 mm
*Z* = 4	

### Data collection

**Table:**

Bruker Kappa APEXII CCD diffractometer'	4674 independent reflections
Radiation source: fine-focus sealed tube	1701 reflections with *I* > 2σ(*I*)
graphite	*R*_int_ = 0.057
ω and φ scans	θ_max_ = 28.4°, θ_min_ = 2.5°
Absorption correction: multi-scan (*SADABS*; Bruker, 2004)	*h* = −15→13
*T*_min_ = 0.978, *T*_max_ = 0.985	*k* = −11→10
14625 measured reflections	*l* = −27→27

### Refinement

**Table:**

Refinement on *F*^2^	Primary atom site location: structure-invariant direct methods
Least-squares matrix: full	Secondary atom site location: difference Fourier map
*R*[*F*^2^ > 2σ(*F*^2^)] = 0.070	Hydrogen site location: inferred from neighbouring sites
*wR*(*F*^2^) = 0.221	H atoms treated by a mixture of independent and constrained refinement
*S* = 1.01	*w* = 1/[σ^2^(*F*_o_^2^) + (0.0923*P*)^2^] where *P* = (*F*_o_^2^ + 2*F*_c_^2^)/3
4674 reflections	(Δ/σ)_max_ < 0.001
266 parameters	Δρ_max_ = 0.30 e Å^−^^3^
0 restraints	Δρ_min_ = −0.14 e Å^−^^3^

### Special details

**Table:**

Geometry. All e.s.d.'s (except the e.s.d. in the dihedral angle between two l.s. planes) are estimated using the full covariance matrix. The cell e.s.d.'s are taken into account individually in the estimation of e.s.d.'s in distances, angles and torsion angles; correlations between e.s.d.'s in cell parameters are only used when they are defined by crystal symmetry. An approximate (isotropic) treatment of cell e.s.d.'s is used for estimating e.s.d.'s involving l.s. planes.
Refinement. Refinement of *F*^2^ against ALL reflections. The weighted *R*-factor *wR* and goodness of fit *S* are based on *F*^2^, conventional *R*-factors *R* are based on *F*, with *F* set to zero for negative *F*^2^. The threshold expression of *F*^2^ > σ(*F*^2^) is used only for calculating *R*-factors(gt) *etc*. and is not relevant to the choice of reflections for refinement. *R*-factors based on *F*^2^ are statistically about twice as large as those based on *F*, and *R*- factors based on ALL data will be even larger.

### Fractional atomic coordinates and isotropic or equivalent isotropic displacement parameters (Å^2^)

**Table:**

	*x*	*y*	*z*	*U*_iso_*/*U*_eq_	
C1	0.6739 (3)	0.0471 (4)	0.09769 (15)	0.0597 (9)	
C2	0.7394 (3)	0.1773 (4)	0.11004 (14)	0.0567 (8)	
C3	0.6655 (3)	0.2848 (4)	0.14155 (14)	0.0553 (8)	
C4	0.6798 (3)	0.4337 (4)	0.16344 (16)	0.0663 (9)	
H4	0.7507	0.4842	0.1595	0.080*	
C5	0.5896 (3)	0.5078 (4)	0.19108 (15)	0.0716 (10)	
H5	0.6000	0.6082	0.2059	0.086*	
C6	0.4831 (3)	0.4337 (5)	0.19703 (17)	0.0770 (11)	
H6	0.4230	0.4842	0.2163	0.092*	
C7	0.4662 (3)	0.2860 (5)	0.17452 (17)	0.0755 (11)	
H7	0.3951	0.2356	0.1777	0.091*	
C8	0.5580 (3)	0.2157 (4)	0.14725 (16)	0.0606 (9)	
C9	0.7056 (3)	−0.1003 (4)	0.06783 (15)	0.0594 (9)	
C10	0.6325 (3)	−0.1789 (5)	0.02351 (19)	0.0823 (11)	
H10	0.5589	−0.1383	0.0111	0.099*	
C11	0.6650 (4)	−0.3161 (5)	−0.0031 (2)	0.0910 (12)	
H11	0.6136	−0.3684	−0.0325	0.109*	
C12	0.7729 (5)	−0.3737 (5)	0.0142 (2)	0.0926 (13)	
H12	0.7963	−0.4649	−0.0045	0.111*	
C13	0.8484 (3)	−0.3000 (5)	0.0588 (2)	0.0807 (11)	
H13	0.9214	−0.3426	0.0712	0.097*	
C14	0.8157 (3)	−0.1631 (4)	0.08506 (17)	0.0687 (10)	
H14	0.8675	−0.1119	0.1146	0.082*	
C15	0.8582 (3)	0.2075 (4)	0.09030 (17)	0.0633 (9)	
C16	0.8850 (3)	0.1954 (4)	0.02908 (16)	0.0647 (9)	
H16	0.9649	0.1998	0.0233	0.078*	
C17	0.8091 (3)	0.1761 (4)	−0.03113 (15)	0.0558 (8)	
C18	0.6996 (3)	0.2448 (4)	−0.04003 (16)	0.0598 (9)	
H18	0.6674	0.2921	−0.0051	0.072*	
C19	0.6376 (3)	0.2438 (4)	−0.1002 (2)	0.0757 (10)	
H19	0.5648	0.2918	−0.1059	0.091*	
C20	0.6835 (4)	0.1720 (5)	−0.15140 (19)	0.0947 (13)	
H20	0.6419	0.1727	−0.1920	0.114*	
C21	0.7894 (4)	0.0995 (5)	−0.1438 (2)	0.0995 (13)	
H21	0.8189	0.0484	−0.1787	0.119*	
C22	0.8522 (3)	0.1024 (4)	−0.08424 (19)	0.0801 (11)	
H22	0.9249	0.0541	−0.0793	0.096*	
C23	0.9520 (3)	0.2436 (4)	0.14166 (17)	0.0627 (9)	
C24	1.0480 (3)	0.3370 (5)	0.1295 (2)	0.0875 (12)	
H24	1.0514	0.3805	0.0885	0.105*	
C25	1.1354 (3)	0.3657 (5)	0.1754 (2)	0.1007 (14)	
H25	1.1977	0.4287	0.1662	0.121*	
C26	1.1321 (4)	0.3016 (6)	0.2357 (2)	0.1003 (14)	
H26	1.1942	0.3174	0.2669	0.120*	
C27	1.0389 (4)	0.2150 (5)	0.2505 (2)	0.0953 (13)	
H27	1.0362	0.1739	0.2920	0.114*	
C28	0.9482 (3)	0.1884 (4)	0.20368 (19)	0.0793 (11)	
H28	0.8834	0.1320	0.2144	0.095*	
N1	0.5641 (3)	0.0706 (4)	0.11907 (15)	0.0738 (9)	
H1N	0.506 (3)	0.001 (5)	0.1187 (18)	0.107 (15)*	

### Atomic displacement parameters (Å^2^)

**Table:**

	*U*^11^	*U*^22^	*U*^33^	*U*^12^	*U*^13^	*U*^23^
C1	0.051 (2)	0.076 (3)	0.053 (2)	0.0087 (17)	0.0059 (15)	0.0111 (17)
C2	0.061 (2)	0.065 (2)	0.0440 (19)	0.0007 (18)	0.0009 (15)	−0.0004 (16)
C3	0.0518 (19)	0.070 (2)	0.0433 (19)	−0.0028 (17)	−0.0002 (14)	0.0064 (17)
C4	0.067 (2)	0.072 (3)	0.060 (2)	−0.0061 (19)	0.0086 (17)	0.0030 (19)
C5	0.081 (3)	0.073 (2)	0.061 (2)	0.008 (2)	0.0041 (19)	−0.0043 (19)
C6	0.064 (2)	0.106 (3)	0.062 (2)	0.025 (2)	0.0081 (18)	0.001 (2)
C7	0.053 (2)	0.104 (3)	0.070 (3)	−0.004 (2)	0.0087 (17)	0.016 (2)
C8	0.059 (2)	0.063 (2)	0.059 (2)	0.0065 (18)	−0.0016 (16)	0.0055 (18)
C9	0.065 (2)	0.061 (2)	0.053 (2)	−0.0056 (18)	0.0043 (17)	0.0070 (17)
C10	0.076 (3)	0.085 (3)	0.083 (3)	−0.013 (2)	−0.010 (2)	0.004 (2)
C11	0.112 (4)	0.076 (3)	0.083 (3)	−0.020 (3)	−0.008 (3)	−0.017 (2)
C12	0.124 (4)	0.072 (3)	0.086 (3)	−0.013 (3)	0.036 (3)	−0.011 (2)
C13	0.084 (3)	0.071 (3)	0.089 (3)	0.000 (2)	0.020 (2)	0.009 (2)
C14	0.076 (3)	0.064 (2)	0.067 (2)	−0.0036 (19)	0.0091 (19)	0.0002 (19)
C15	0.058 (2)	0.074 (2)	0.058 (2)	0.0023 (16)	0.0013 (17)	−0.0049 (18)
C16	0.060 (2)	0.073 (2)	0.061 (2)	−0.0035 (17)	0.0036 (18)	−0.0031 (18)
C17	0.0472 (19)	0.068 (2)	0.051 (2)	−0.0121 (16)	−0.0044 (15)	−0.0015 (17)
C18	0.066 (2)	0.060 (2)	0.052 (2)	−0.0105 (17)	−0.0013 (17)	−0.0002 (16)
C19	0.079 (2)	0.072 (3)	0.073 (3)	−0.0073 (19)	−0.012 (2)	0.010 (2)
C20	0.115 (4)	0.113 (3)	0.052 (3)	−0.021 (3)	−0.011 (2)	−0.007 (2)
C21	0.100 (3)	0.136 (4)	0.063 (3)	−0.016 (3)	0.008 (2)	−0.032 (3)
C22	0.065 (2)	0.100 (3)	0.076 (3)	0.000 (2)	0.009 (2)	−0.022 (2)
C23	0.053 (2)	0.071 (2)	0.062 (2)	0.0034 (17)	−0.0091 (17)	−0.0047 (18)
C24	0.063 (2)	0.128 (4)	0.071 (3)	−0.006 (2)	0.002 (2)	−0.018 (2)
C25	0.058 (3)	0.152 (4)	0.091 (3)	−0.008 (2)	−0.002 (2)	−0.023 (3)
C26	0.062 (3)	0.146 (4)	0.089 (4)	0.014 (3)	−0.016 (2)	−0.030 (3)
C27	0.091 (3)	0.129 (4)	0.063 (3)	0.005 (3)	−0.011 (2)	−0.012 (2)
C28	0.076 (3)	0.092 (3)	0.067 (3)	−0.004 (2)	−0.013 (2)	0.000 (2)
N1	0.060 (2)	0.083 (2)	0.078 (2)	−0.0147 (18)	0.0016 (16)	0.0084 (18)

### Geometric parameters (Å, °)

**Table:**

C1—C2	1.370 (4)	C15—C16	1.326 (4)
C1—N1	1.376 (4)	C15—C23	1.481 (5)
C1—C9	1.479 (4)	C16—C17	1.468 (4)
C2—C3	1.446 (4)	C16—H16	0.9300
C2—C15	1.470 (4)	C17—C18	1.386 (4)
C3—C4	1.377 (4)	C17—C22	1.391 (4)
C3—C8	1.380 (4)	C18—C19	1.381 (5)
C4—C5	1.376 (4)	C18—H18	0.9300
C4—H4	0.9300	C19—C20	1.365 (5)
C5—C6	1.392 (5)	C19—H19	0.9300
C5—H5	0.9300	C20—C21	1.362 (5)
C6—C7	1.374 (5)	C20—H20	0.9300
C6—H6	0.9300	C21—C22	1.375 (5)
C7—C8	1.372 (4)	C21—H21	0.9300
C7—H7	0.9300	C22—H22	0.9300
C8—N1	1.393 (4)	C23—C28	1.370 (5)
C9—C10	1.372 (5)	C23—C24	1.404 (5)
C9—C14	1.392 (4)	C24—C25	1.345 (5)
C10—C11	1.377 (5)	C24—H24	0.9300
C10—H10	0.9300	C25—C26	1.365 (6)
C11—C12	1.352 (5)	C25—H25	0.9300
C11—H11	0.9300	C26—C27	1.358 (5)
C12—C13	1.371 (5)	C26—H26	0.9300
C12—H12	0.9300	C27—C28	1.380 (5)
C13—C14	1.372 (5)	C27—H27	0.9300
C13—H13	0.9300	C28—H28	0.9300
C14—H14	0.9300	N1—H1N	0.89 (4)
			
C2—C1—N1	108.4 (3)	C2—C15—C23	118.2 (3)
C2—C1—C9	130.3 (3)	C15—C16—C17	130.5 (3)
N1—C1—C9	121.4 (3)	C15—C16—H16	114.8
C1—C2—C3	106.8 (3)	C17—C16—H16	114.8
C1—C2—C15	126.7 (3)	C18—C17—C22	117.7 (3)
C3—C2—C15	126.3 (3)	C18—C17—C16	122.1 (3)
C4—C3—C8	117.8 (3)	C22—C17—C16	119.8 (3)
C4—C3—C2	134.2 (3)	C19—C18—C17	120.8 (3)
C8—C3—C2	108.0 (3)	C19—C18—H18	119.6
C5—C4—C3	120.2 (3)	C17—C18—H18	119.6
C5—C4—H4	119.9	C20—C19—C18	119.8 (4)
C3—C4—H4	119.9	C20—C19—H19	120.1
C4—C5—C6	120.5 (3)	C18—C19—H19	120.1
C4—C5—H5	119.8	C21—C20—C19	120.9 (4)
C6—C5—H5	119.8	C21—C20—H20	119.5
C7—C6—C5	120.3 (3)	C19—C20—H20	119.5
C7—C6—H6	119.9	C20—C21—C22	119.4 (4)
C5—C6—H6	119.9	C20—C21—H21	120.3
C8—C7—C6	117.6 (3)	C22—C21—H21	120.3
C8—C7—H7	121.2	C21—C22—C17	121.3 (4)
C6—C7—H7	121.2	C21—C22—H22	119.3
C7—C8—C3	123.6 (3)	C17—C22—H22	119.3
C7—C8—N1	129.8 (3)	C28—C23—C24	116.8 (3)
C3—C8—N1	106.6 (3)	C28—C23—C15	121.4 (3)
C10—C9—C14	117.7 (3)	C24—C23—C15	121.8 (3)
C10—C9—C1	123.6 (3)	C25—C24—C23	122.0 (4)
C14—C9—C1	118.6 (3)	C25—C24—H24	119.0
C9—C10—C11	121.9 (4)	C23—C24—H24	119.0
C9—C10—H10	119.0	C24—C25—C26	119.6 (4)
C11—C10—H10	119.0	C24—C25—H25	120.2
C12—C11—C10	119.0 (4)	C26—C25—H25	120.2
C12—C11—H11	120.5	C27—C26—C25	120.5 (4)
C10—C11—H11	120.5	C27—C26—H26	119.7
C11—C12—C13	121.1 (4)	C25—C26—H26	119.7
C11—C12—H12	119.4	C26—C27—C28	119.7 (4)
C13—C12—H12	119.4	C26—C27—H27	120.2
C12—C13—C14	119.6 (4)	C28—C27—H27	120.2
C12—C13—H13	120.2	C23—C28—C27	121.3 (4)
C14—C13—H13	120.2	C23—C28—H28	119.4
C13—C14—C9	120.5 (4)	C27—C28—H28	119.4
C13—C14—H14	119.7	C1—N1—C8	110.1 (3)
C9—C14—H14	119.7	C1—N1—H1N	126 (2)
C16—C15—C2	122.4 (3)	C8—N1—H1N	124 (2)
C16—C15—C23	119.4 (3)		
			
N1—C1—C2—C3	0.9 (3)	C3—C2—C15—C16	−120.7 (4)
C9—C1—C2—C3	−178.4 (3)	C1—C2—C15—C23	−122.5 (3)
N1—C1—C2—C15	−174.5 (3)	C3—C2—C15—C23	63.0 (4)
C9—C1—C2—C15	6.2 (5)	C2—C15—C16—C17	10.5 (6)
C1—C2—C3—C4	−177.6 (3)	C23—C15—C16—C17	−173.2 (3)
C15—C2—C3—C4	−2.2 (6)	C15—C16—C17—C18	35.3 (5)
C1—C2—C3—C8	0.1 (3)	C15—C16—C17—C22	−152.4 (4)
C15—C2—C3—C8	175.5 (3)	C22—C17—C18—C19	−2.0 (4)
C8—C3—C4—C5	1.2 (4)	C16—C17—C18—C19	170.4 (3)
C2—C3—C4—C5	178.8 (3)	C17—C18—C19—C20	1.2 (5)
C3—C4—C5—C6	−0.2 (5)	C18—C19—C20—C21	0.8 (6)
C4—C5—C6—C7	−0.9 (5)	C19—C20—C21—C22	−1.8 (6)
C5—C6—C7—C8	0.9 (5)	C20—C21—C22—C17	0.9 (6)
C6—C7—C8—C3	0.2 (5)	C18—C17—C22—C21	1.0 (5)
C6—C7—C8—N1	−177.8 (3)	C16—C17—C22—C21	−171.6 (3)
C4—C3—C8—C7	−1.3 (5)	C16—C15—C23—C28	−148.1 (3)
C2—C3—C8—C7	−179.4 (3)	C2—C15—C23—C28	28.3 (5)
C4—C3—C8—N1	177.1 (3)	C16—C15—C23—C24	32.6 (5)
C2—C3—C8—N1	−1.0 (3)	C2—C15—C23—C24	−150.9 (3)
C2—C1—C9—C10	−138.1 (4)	C28—C23—C24—C25	3.1 (5)
N1—C1—C9—C10	42.6 (5)	C15—C23—C24—C25	−177.6 (3)
C2—C1—C9—C14	41.5 (5)	C23—C24—C25—C26	0.5 (6)
N1—C1—C9—C14	−137.8 (3)	C24—C25—C26—C27	−3.0 (7)
C14—C9—C10—C11	0.6 (5)	C25—C26—C27—C28	1.7 (6)
C1—C9—C10—C11	−179.8 (3)	C24—C23—C28—C27	−4.4 (5)
C9—C10—C11—C12	−1.1 (6)	C15—C23—C28—C27	176.3 (3)
C10—C11—C12—C13	1.8 (6)	C26—C27—C28—C23	2.1 (6)
C11—C12—C13—C14	−2.0 (6)	C2—C1—N1—C8	−1.6 (4)
C12—C13—C14—C9	1.4 (5)	C9—C1—N1—C8	177.8 (3)
C10—C9—C14—C13	−0.7 (5)	C7—C8—N1—C1	179.9 (3)
C1—C9—C14—C13	179.6 (3)	C3—C8—N1—C1	1.6 (4)
C1—C2—C15—C16	53.8 (5)		

### Hydrogen-bond geometry (Å, °)

**Table:**

Cg1 and Cg2 are the centroids of the N1/C1/C2/C3/C8 and C3–C8 rings, respectively.

**Table:**

*D*—H···*A*	*D*—H	H···*A*	*D*···*A*	*D*—H···*A*
C18—H18···Cg1	0.93	2.92	3.562 (2)	127
C20—H20···Cg2^i^	0.93	2.92	3.825 (2)	164

Symmetry codes:  (i) *x*, −*y*−1/2, *z*−3/2.

## References

[bb1] Allen, F. H., Kennard, O., Watson, D. G., Brammer, L., Orpen, A. G. & Taylor, R. (1987). *J. Chem. Soc. Perkin Trans. 2*, pp. S1–19.

[bb2] Bruker (2004). *APEX2*, *SAINT*, *XPREP* and *SADABS* Bruker AXS Inc., Madison, Wisconsin, USA.

[bb3] Cheng, C. H., Bhuvaneswari, S. & Jeganmohan, M. (2007). *Chem. Eur. J.* pp. 8285–8293. 10.1002/chem.200700589 17721891

[bb4] Farrugia, L. J. (1997). *J. Appl. Cryst.* **30**, 565.

[bb5] Ferrer, C., Amijs, C. H. M. & Echavarren, A. M. (2007). *Chem. Eur. J.* pp. 1358–1373. 10.1002/chem.200601324 17206736

[bb6] Nair, V., Ros, S., Jayan, C. N. & Pillai, B. S. (2004). *Tetrahedron* **60**, 1959–1982.

[bb7] NizamMohideen, M., Bhaskar, G. & Perumal, P. T. (2010*a*). *Acta Cryst.* E**66**, o2506. 10.1107/S1600536810034707 PMC2983385 21587502

[bb8] NizamMohideen, M., Bhaskar, G. & Perumal, P. T. (2010*b*). *Acta Cryst.* E**66**, o2514. 10.1107/S1600536810034719 PMC2983220 21587508

[bb9] Ritleng, V., Sirlin, C. & Pfeffer, M. (2002). *Chem. Rev.* **102**, 1731–1769. 10.1021/cr0104330 11996548

[bb10] Sakai, N., Annaka, K., Fujita, A., Sato, A. & Konakahara, T. (2008). *J. Org. Chem* **73**, 4160–4165. 10.1021/jo800464u 18442294

[bb11] Sakai, N., Annaka, K. & Konakahara, T. (2006). *Tetrahedron Lett.* **47**, 631–634.

[bb12] Sheldrick, G. M. (2008). *Acta Cryst.* A**64**, 112–122. 10.1107/S0108767307043930 18156677

[bb13] Spek, A. L. (2009). *Acta Cryst* D**65**, 148–155. 10.1107/S090744490804362X PMC2631630 19171970

[bb14] Sundberg, R. J. (1996). *Indoles.* London: Academic Press.

